# D-Serine Contributes to Seizure Development via ERK Signaling

**DOI:** 10.3389/fnins.2019.00254

**Published:** 2019-03-26

**Authors:** Tie Ma, Yin Wu, Beibei Chen, Wenjuan Zhang, Lang Jin, Chenxi Shen, Yazhou Wang, Yonghong Liu

**Affiliations:** ^1^Department of Neurology, Xijing Hospital, Air Force Military Medical University, Xi’an, China; ^2^Department of Neurology, The Seventh Medical Center of PLA General Hospital, Beijing, China; ^3^Department of Pharmacy, Xi’an High-tech Hospital, Xi’an, China; ^4^Department of Neurobiology and Institute of Neurosciences, School of Basic Medicine, Air Force Medical University, Xi’an, China

**Keywords:** D-serine, serine racemase, astrocyte, epilepsy, ERK, hippocampus

## Abstract

A seizure is one of the leading neurological disorders. NMDA receptor-mediated neuronal excitation has been thought to be essential for epileptogenesis. As an endogenous co-agonist of the NMDA receptor, D-serine has been suggested to play a role in epileptogenesis. However, the underlying mechanisms remain unclear. In the current study, we investigated the effects of antagonizing two key enzymes in D-serine metabolism on the development of seizures and the downstream signaling. Our results showed that serine racemase (SR), a key enzyme in regulating the L-to-D-serine conversion, was significantly up-regulated in hippocampal astrocytes in rats and patients who experienced seizure, in comparison with control rats and patients. L-aspartic acid β-hydroxamate (LaaβH), an inhibitor of SR, significantly prolonged the latencies of seizures, shortened the durations of seizures, and decreased the total EEG power in rats. In contrast, D-amino acid oxidase inhibitor 5-chlorobenzo[d]isoxazol-3-ol (CBIO), which can increase D-serine levels, showed the opposite effects. Furthermore, our data showed that LaaβH and CBIO significantly affected the phosphorylation of Extracellular Signal-regulated Kinase (ERK). Antagonizing or activating ERK could significantly block the effects of LaaβH/CBIO on the occurrence of seizures. In summary, our study revealed that D-serine is involved in the development of epileptic seizures, partially through ERK signaling, indicating that the metabolism of D-serine may be targeted for the treatment of epilepsy.

## Introduction

Epilepsy includes a group of neurological disorders characterized by loss of neurons and spontaneous occurrence of seizures. Although the underlying mechanisms remain to be fully elucidated, it has been widely accepted that *N*-methyl-D-aspartate receptor (NMDAR)-mediated neuronal hyperexcitation is essential for the development of epilepsy (Parsons and Raymond, 2014; Swanger et al., 2016; Rothan et al., 2017). Recently, increasing evidence has emerged that astrocytes are also involved in the development of epilepsy and seizure-induced excitotoxicity (Ding et al., 2007; Gomez-Gonzalo et al., 2010; Clasadonte et al., 2013). However, the mechanism through which astrocytes contribute to the hyperexcitation of neurons during epilepsy remains unclear.

D-serine is an endogenous co-agonist of the NMDAR and is expressed in most regions of the mammalian brain. D-serine acts as an allosteric modulator of the NMDAR channel by binding to the glycine site of the NMDAR (Kleckner and Dingledine, 1988; Schell et al., 1995). In both astrocytes and neurons, D-serine is converted from L-serine by serine racemase (SR). The astrocytic D-serine is shuttled to neurons, and the neuronal D-serine is released upon depolarization to activate NMDARs (Ehmsen et al., 2013; Wolosker et al., 2016). By agonizing NMDAR-mediated responses, D-serine plays important roles in the enhancement of synaptic functions and the maintenance of long term potentiation (Balu et al., 2013; Le Bail et al., 2015).

Because of its ability to modulate the excitability of neurons, D-serine has been suggested to play roles in multiple neurological conditions (Kew et al., 2000; Ballard et al., 2002; Bado et al., 2011). Particularly in epileptic animals, the levels of D-serine have been shown to be reduced in the forebrain, and this change of D-serine has been thought to contribute to cognitive dysfunction (Klatte et al., 2013). Our previous study showed that D-serine is rapidly upregulated in degenerating GABAergic neurons in the hippocampus (Liu et al., 2009). Because NMDAR antagonists have been suggested to be effective for anticonvulsive treatment, it is possible that the pharmacological manipulation of D-serine could affect the development of seizures; this hypothesis remains poorly investigated.

In the present study, we addressed this question by using two compounds that interfere with the metabolism of D-serine in a lithium-pilocarpine-induced epileptic seizure model and by examining the expression levels of SR in patients who suffered seizures.

## Materials and Methods

### Animals

Male Sprague–Dawley rats (180–200 g) were used. Animals were maintained on a 12 h light/dark cycle at room temperature (55% humidity), with a normal diet of rat chow *ad libitum*. All animal procedures were approved by the Air Force Military Medical University Animal Care Committee and were performed in accordance with the National Institutes of Health Guide for the Care and Use of Laboratory Animals.

### Patient Samples

The experimental protocols involving patient samples were approved by the ethics committee of the Air Force Medical University, and informed consent was provided by all patients. All experiments and methods using human samples were approved by and performed in accordance with The Code of Ethics of the World Medical Association and the guidelines issued by the ethics committee of the Air Force Medical University.

Thirteen patients aged 28.35 ± 2.38 years, who were diagnosed with refractory temporal lobe epilepsy (TLE) with unilateral hippocampal sclerosis, as detected by magnetic resonance imaging (MRI), were included in the present study. Patients who did not achieve seizure control under adequate treatment using at least two first-line anti-epilepsy drugs were defined as being medically refractory. All patients were subjected to extensive presurgical evaluations, including high-resolution 3.0 T MRI, prolonged non-invasive video-EEG recording, and neuropsychological testing. Right hippocampal resections were performed in 7 cases, and left hippocampal resections were performed in 6 cases. After surgery, hippocampal tissue was fixed overnight in 4% formalin and cryoprotected by incubation in 30% sucrose in 0.01 M phosphate buffer overnight at 4°C. Frozen sections were cut using a cryostat (CM1900, Leica, Heidelberger, Germany) and mounted on slides coated with polylysine.

Five patients (3 men and 2 women), with a mean age of 29.7 ± 1.74 years, who suffered traumatic brain injury were included as control patients in this study. All patients underwent surgery within 6 h post-trauma, and parts of the hippocampus were surgically removed from each patient. All specimens were post-fixed and cryoprotected as described above.

### Intracerebral Ventricular Cannula Implantation and Electrode Placement

For drug delivery to the brain, a unilateral 23-gauge single guide cannula (WRD, Shenzhen, China) was implanted in each rat. Under sterile conditions, an incision was made along the midline of the scalp. The cannula guide was inserted at -0.8 mm from Bregma, ±1.5 mm lateral from the midline, and -3.5 mm from dura, as described previously (Lubin and Sweatt, 2007). For EEG recording, four cortical electrodes were placed as follows: one in the CA1 region (coordinates vs. bregma: -3 mm anteroposterior, -1.5 mm lateral, -3.3 mm depth), one in the left frontal lobe (+2.0 mm anteroposterior, -3.0 mm lateral, -1.5 mm depth), one in each of the left and right occipital lobes (+2.0 mm anteroposterior, -3 mm lateral), and a grounding wire. Two anchoring screws were placed in the remaining distal area. The screw-electrode complex was fixed with cold-curing dental cement, and the incision site was sealed with absorbable sutures. After surgery, the rats were left to recover in a temperature-controlled heating element. Animals were habituated to the cannula and electrodes for 7 days before experiments.

### Drug Treat and Seizure Induction

To detect the roles of the NMDA receptor and D-serine in epilepsy, rats were pretreated with one of the following for half an hour before the induction of seizures through the administration of pilocarpine: the NMDAR antagonist MK801 (0.6 mg kg^-1^, i.p.; Sigma); a competitive SR inhibitor, LAAβH (100 mg ml^-1^. i.c.v.; 5 μl, Sigma); a DAAO inhibitor, CBIO (1 mg ml^-1^, i.c.v.; 5 μl, Sigma), which can increase the levels of SR; or saline (5 μl i.c.v) as a control. To manipulate ERK activity, U46619 (2 mg/ml, i.c.v.; 5 μl, R & D) or PD98059 (300 nmol, i.c.v.; 5 μl, Tocris) was administered 30 min before seizure induction.

Seizure-induction process was performed as described in our previous study (Liu et al., 2009). A total of 127 mg/kg lithium chloride (Sigma) was intraperitoneally (i.p.) administered 18–20 h before pilocarpine (50 mg/kg, Sigma) treatment. Video monitoring was performed using a SOLAR3000N system. The EEG signals from individual electrodes were recorded and analyzed using Acknowledge software (BIOPAC Systems Inc., United States). EEG discharges were defined as repetitive spikings (amplitude ≥3 times baseline) lasting more than 15 s. Video data were analyzed to correlate EEG activity with behavioral seizure activity, which was scored according to the Racine scale (stage 1, mouth and facial movements; stage 2, head nodding; stage 3, forelimb clonus; stage 4, rearing; stage 5, rearing and falling) (Racine, 1972). Rats showing stage 4 or 5 seizures for 1 h were injected with diazepam (10 mg/kg, i.p.) to terminate status epilepticus and used for subsequent experiments. To calculate the “latency to SE” or the “latency to onset of stage 3 seizures,” the starting time was considered to be the time point of the pilocarpine injection.

The rats were sacrificed at various time points (ranging from 24 to 48 h) after the termination of status epilepticus (*n* = 19 for each group). For Western blot analysis, the hippocampus and neocortex were quickly dissected on ice and stored at -80°C. For immunohistochemistry analysis, rats were anesthetized with chloral hydrate and perfused with 4% paraformaldehyde.

### High Performance Liquid Chromatography (HPLC)

Hippocampal samples were extracted by HPLC-grade acetonitrile (Sigma) and aliquots were dried in a speed vacuum system. Stock solutions of D-serine (2 mg/ml, Sigma) and ^13^C3, ^15^N DL-serine (internal standard, 2 mg/ml, Cambridge Isotope Labs) were prepared in HPLC-grade water. Calibration standard (CS) and quality control (QC) samples were prepared by serial dilution of D-serine stock solution with methanol/water (80/20). CS and QC samples were extracted with 4 volumes of acetonitrile, and the supernatants evaporated to dryness by nitrogen. For HPLC analysis, aliquots of tissue samples, CS and QC samples were reconstituted in LC/MS (95/5 methanol/watert, 10 mM ammonium formate and 0.1% formic acid). Internal standard DL-serine was added to the LC/MS to a concentration of 0.5 mg/ml. Samples were run by a Shimadzu LC-10AD liquid chromatography system equipped with a degasser and a CTC Analytics HTS PAL Autosampler and an Astec Chirobiotic-T column (2.1 × 250 mm, 5 mm) at a flow rate of 0.5 ml per minute. The mobile phase constituted a 95/5 mixture of phase A (methanol) and phase B (water with 10 mM ammonium formate + 0.1% formic acid). Quantitation was achieved by MS/MS detection in positive ion mode. Detection was performed in the selected reaction monitoring mode, monitoring the transition of m/z 106.1 to 60.1 for D-serine and m/z 110 to 63.1 for the internal standard.

### Immunohistochemistry

Frozen sections (20-μm thick) were prepared for the detection of SR expression by immunofluorescent staining. Tissue sections were incubated with the following primary antibodies at 4°C overnight: rabbit anti-SR antibody (1:100, Santa), mouse anti-GFAP antibody (1:1000, Abcam), rabbit anti-SR antibody (1:500, Abcam), rabbit anti-D-serine antibody (1:1000, Abcam), and mouse anti-NeuN antibody (1:1000, Chemicon). After rinsing with PBS, the sections were incubated with corresponding secondary antibodies conjugated to Alexa Fluor 594 or Alexa Fluor 488 (Jackson Immunoresearch) for 2–4 h at room temperature and protected from light. For the staining of SR, a competitive peptide was used as a control to ensure the specificity of the anti-SR antibody. The nuclei were counterstained by DAPI (Sigma, St Louis, MO, United States).

### Western Blotting

Protein extract from brain tissues was separated by SDS-PAGE and transferred to nitrocellulose membranes. After blocking, the membranes were incubated with the following primary antibodies at 4°C overnight: mouse anti-p-AKT (1:1,000, Cell Signaling), mouse anti-AKT (1:1,000, Cell Signaling), rabbit anti-p-JNK (1:500, Cell Signaling), JNK (1:600, Cell Signaling), rabbit anti-p-ERK (targeting the phosphorylation sites Thr202 and Tyr204, 1:1000, Cell Signaling), mouse anti-ERK (1:500, Cell Signaling), mouse anti-β-actin (1:600; Santa Cruz Biotechnology), and rabbit anti-GAPDH (1:1000; Cell Signaling). The membranes were then washed and incubated with peroxidase-conjugated secondary antibodies for 1 h at room temperature. Specific bands were visualized using the ECL system and were analyzed using Image J.

### Image Collection and Statistical Analysis

All slides were examined using a confocal laser scanning microscope (Fluoview 1000; Olympus, Tokyo, Japan). Digital images were captured using the Fluoview application software (Olympus, Tokyo, Japan). All experiments were replicated for at least 3 times. For the behavior study, at least 5 rats were included in each group. The data are presented as the mean ± SE and were analyzed using one-way ANOVA with Tukey’s *post hoc* test or Student’s *t*-test with SPSS 13.0 software. *P* values less than 0.05 were defined as being statistically significant.

## Results

### Serine Racemase (SR) Is Upregulated in the Hippocampi of Epileptic Rats and Patients

**FIGURE 1:**
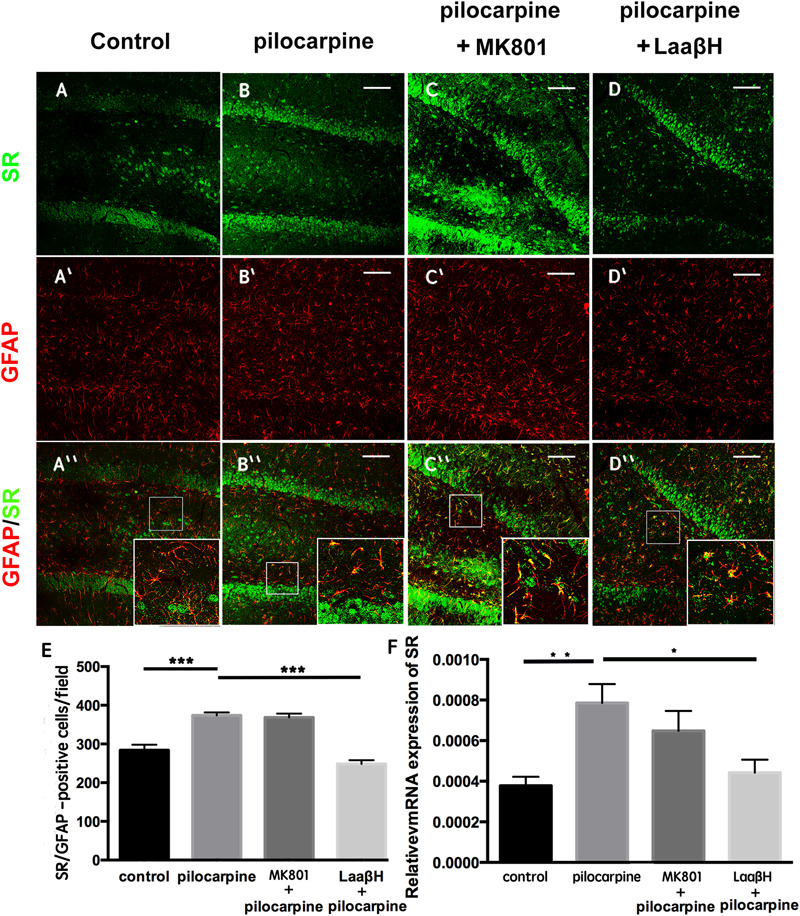
Expression of SR in astrocytes after seizure induction in rats. **(A–D)** Double immunostaining of GFAP/SR in the hippocampi of control rats **(A,A’,A”)**, pilocarpine treated rats **(B,B’,B”)**, pilocarpine + MK801 treated rats **(C,C’,C”)**, and pilocarpine + LaaβH treated rats **(D, D’, D”)**. **(E)** Quantification of SR/GFAP-positive cells in the hippocampi of rats treated with pilocarpine, MK801, pilocarpine + MK801, and pilocarpine + LaaβH. *N* = 3–5 rats per group. **(F)** Real-time RT-PCR of SR in the hippocampi of rats treated with pilocarpine, MK801, pilocarpine + MK801, and pilocarpine + LaaβH. *N* = 3 rats per group. The expression level of SR was upregulated after seizure induction. MK801 had no significant effects on the induction of SR, and LaaβH could inhibit the up-regulation of SR by seizures. ^∗^*P* < 0.05, ^∗∗^*P* < 0.01, ^∗∗∗^*P* < 0.001. Bars = 50 μm.

To explore the expression of D-serine in rats that experience epilepsy, we adopted a well-established pilocarpine-induction model, which is known to be highly isomorphic with human TLE (Furman, 2013). Because previous studies have reported that epilepsy induces astrocyte activation and that L-serine (the precursor of D-serine) is primarily synthesized by astrocytes, we first examined the expression of D-serine after seizure induction by double immunostaining for GFAP and SR, a key enzyme that transforms L-serine into D-serine (Wolosker et al., 1999). We focused on the time points of 48 h post pilocarpine treatment when reactive astrocytes began to appear. The results showed that, in the hippocampus, SR was expressed in both neurons and astrocytes. No significant change of SR/NeuN-positive cells was found between control and epileptic rats (Supplementary Figure S1). More SR/GFAP- and GFAP-positive cells were detected in the hippocampi of rats which experienced seizures (Figure 1A,B,E and Supplementary Figure S2). MK801, the NMDA receptor antagonist, showed no significant effects on the number of SR/GFAP-positive cells in the seizure attacked rats (Figure 1C,E). However, administration of L-aspartic acid β-hydroxamate (LaaβH, a competitive SR inhibitor) (Hoffman et al., 2009) significantly reduced the number of SR/GFAP-positive cells in rats treated with pilocarpine (Figure 1D,E). Similar changes of SR expression in rats treated by pilocarpine only or by pilocarpine plus LaaβH were confirmed by real-time RT-PCR (Figure 1F). These data indicated that the expression of SR is up-regulated mainly by astrocytes in the hippocampus after seizure induction.

**FIGURE 2:**
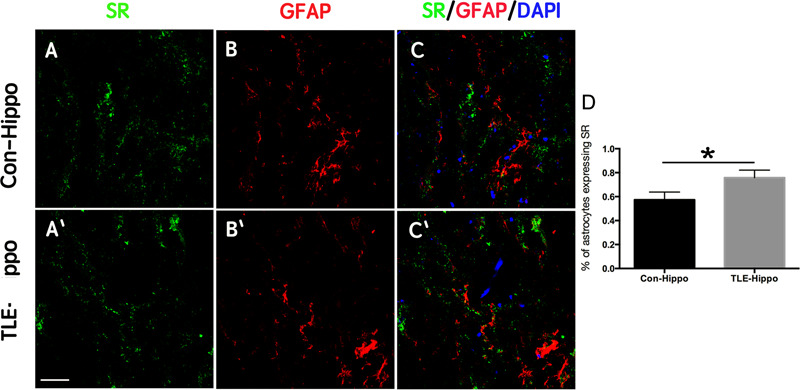
Expression of SR in TLE patients. **(A–C)** Double-immunostaining of GFAP/SR in the hippocampi of control and TLE patients. **(D)** Quantification of astrocytes expressing SR in the hippocampi of control and TLE patients. The expression of SR was up-regulated in the hippocampi of TLE patients. ^∗^*P* < 0.05. Bars = 50 μm.

We next examined the expression of SR in the hippocampi of TLE patients using immunohistochemistry. Similar to what was observed in rats, SR and GFAP were up-regulated in the hippocampi of TLE patients (Figure 2 and Supplementary Figure S3). In particular, the percentage SR-positive astrocytes increased from 57.24 ± 7.73% in the hippocampi of control patients to 75.50 ± 8.27% in the hippocampi of 7 epilepsy patients (Figure 2). These data indicated that D-serine may also be increased in patients who experience seizures.

**FIGURE 3:**
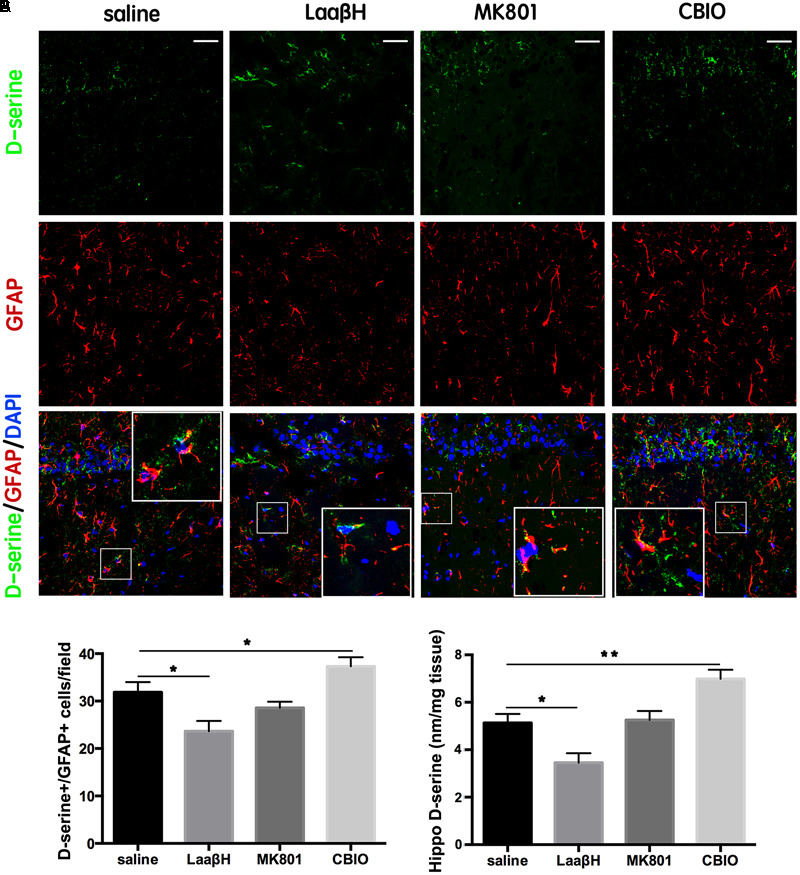
Effects of MK801, LaaβH and CBIO on the expression of D-serine in the hippocampi of rats. **(A)** Double-immunostaining for GFAP/ D-serine in the hippocampi of rats that experienced seizures and were treated with saline, LaaβH, MK801, or CBIO. **(B)** Quantification of the GFAP/D-serine double positive cells shown in **(A)**. **(C)** HPLC analysis of D-serine levels in hippocampus of rats treated by saline, LaaβH, MK801, or CBIO. Notice the decrease of D-serine in the LaaβH-treated rats and increase of D-serine in the CBIO-treated rats. Inserts are magnified double positive cells. ^∗^*P* < 0.05, ^∗∗^*P* < 0.01. Bars = 50 μm.

To test whether the level of D-serine could be affected by pharmacologically interfering with its metabolism, we treated rats with saline, LaaβH, MK801, or 5-chlorobenzo[d]isoxazol-3-ol (CBIO), a compound which can increase the levels of D-serine (Ferraris et al., 2008) and performed immunohistochemistry and high performance liquid Chromatography (HPLC). In comparison with saline control, LaaβH treatment resulted in fewer D-serine/GFAP positive cells in the hippocampus, while CBIO treatment led to more D-serine/GFAP-positive cells (Figure 3A,B). HPLC analysis showed that LaaβH treatment significantly decreased while CIBO treatment significantly increased the levels of D-serine in hippocampus (Figure 3C). These data indicated that LaaβH and CBIO injection could effectively affect the levels of D-serine in the brain.

### Interfering With D-Serine Levels Affects Seizure-Associated Behaviors and Electroencephalograph (EEG) Recordings in Rats

**FIGURE 4:**
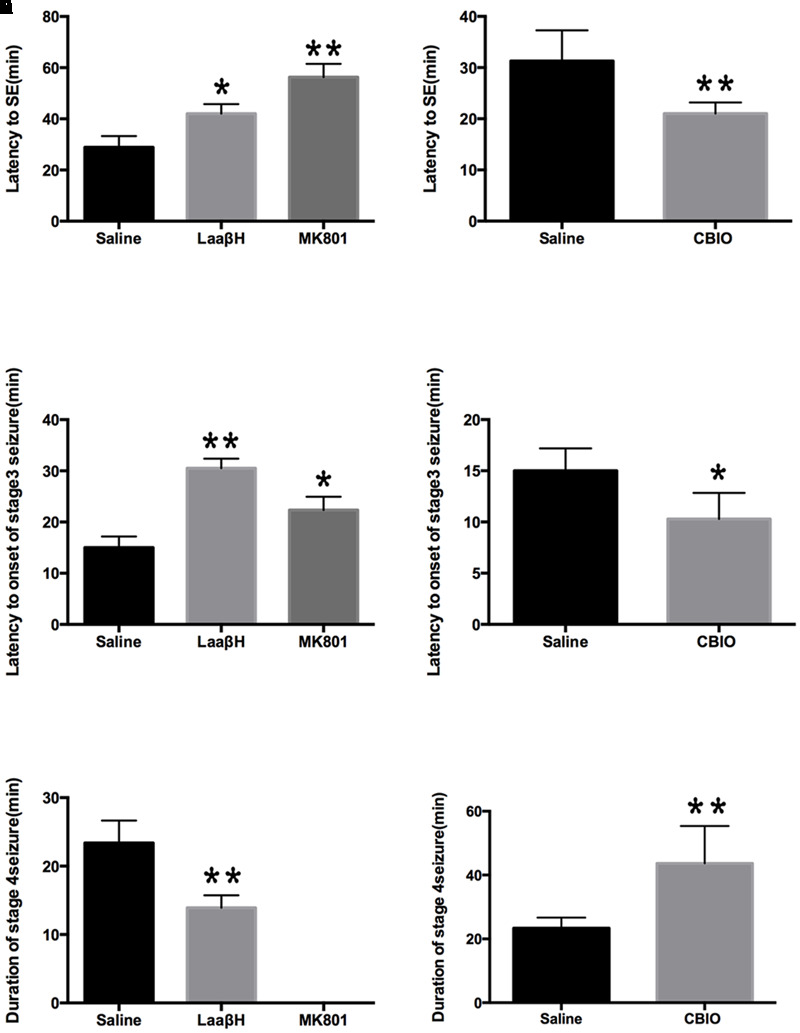
Effects of MK801, LaaβH and CBIO on the on the development of seizures. **(A,B)** Effects of LaaβH and MK801 on the latency to stage 3 seizures. **(C)** Effects of LaaβH and MK801 on the duration of stage 4 seizures. **(D,E)** Effects of CBIO on the latency to stage 3 seizures. **(F)** Effects of CBIO on the duration of stage 4 seizures. *N* = 5 rats per group. LaaβH could significantly inhibit the development of seizures, while CBIO could significantly stimulate the development of seizures. ^∗^*P* < 0.05, ^∗∗^*P* < 0.01.

We next tested whether D-serine was involved in pilocarpine-induced epileptogenesis. As expected, the NMDAR antagonist MK801 significantly prolonged the latencies to seizure occurrence and to stage 3 seizures, confirming the role of NMDAR overactivation in seizure genesis. LaaβH treatment, which could lower the level of D-serine, significantly increased the latencies to seizure occurrence and to stage 3 seizures (Figure 4A,B). In contrast, CBIO significantly shortened the latencies to seizure occurrence and to stage 3 seizures (*n* = 17) (Figure 4D,E). Regarding the duration of stage 4 seizures, MK801 significantly reduced the time of duration as expected (Figure 4C). Rats treated with LaaβH showed a moderate decrease in seizure duration, while CBIO significantly increased the duration of seizures (Figure 4F). These data indicated that interfering with D-serine levels could affect the pilocarpine-induced seizures.

**FIGURE 5:**
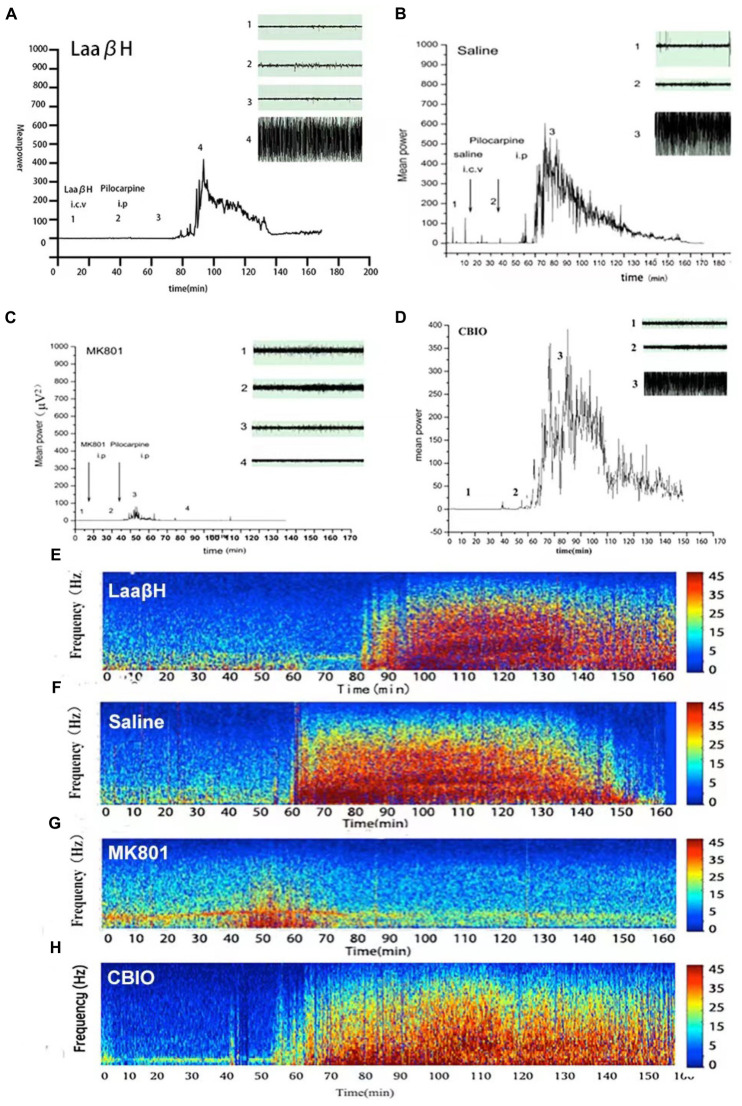
Effects of MK801, LaaβH, and CBIO on the EEG recordings. **(A–D)** Mean power of EEG recordings in rats treated with LaaβH **(A)**, saline **(B)**, MK801 **(C)**, and CBIO **(D)**. **(E–H)** Representative frequency images of EEG recordings in rats treated with LaaβH **(E)**, saline **(F)**, MK801 **(G)**, and CBIO **(H)**. *N* = 7–9 rats per group. Compared with the saline control, LaaβH could prolong the onset of seizure occurrence and reduce the mean power of the EEG, while CBIO could shorten the onset of seizure induction and increase the mean power of the EEG.

We next performed continuous video EEG recordings that commenced 1 h before pilocarpine administration and continued for 72 h after SE. The latency to the first epileptiform discharge in EEG, the latency to the first seizure, and the duration of the first seizure were analyzed. The results showed that the latency to the onset of SE was significantly prolonged in LaaβH treated rats (43.33 ± 2.35 min, *n* = 9) compared with the control group (37.00 ± 1.74 min, *n* = 7. Figure 5A–D). In addition, MK801 and LaaβH dramatically reduced the mean power of the EEG, while CBIO significantly increased the mean power of the EEG (Figure 5E–H). These data indicated that modulating the levels of D-serine could affect not only the behavior of animals but also the EEG activity of seizures.

### Interfering With D-Serine Levels Affects the Phosphorylation of ERK

**FIGURE 6:**
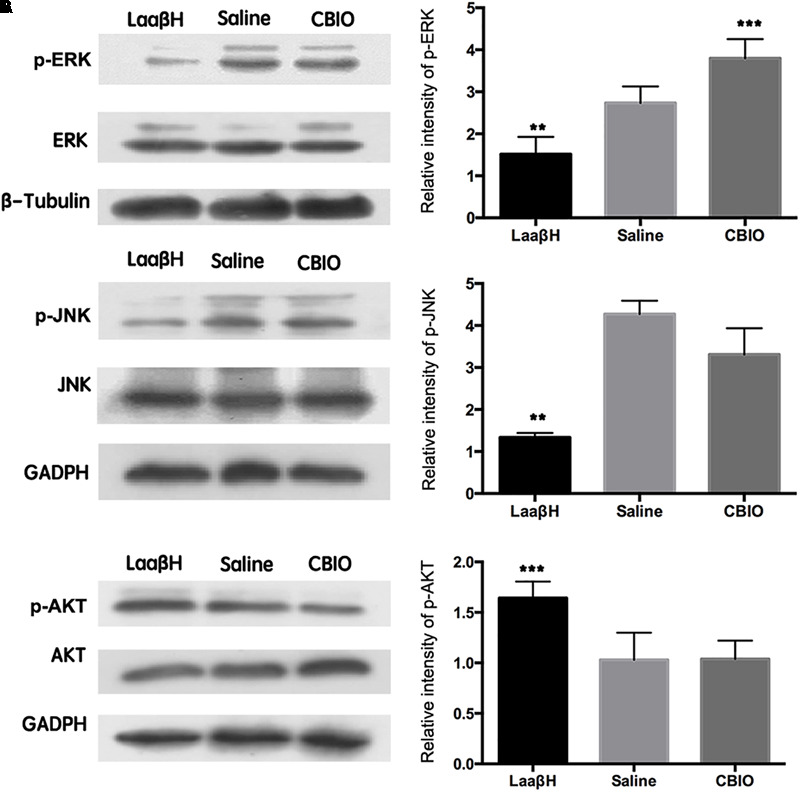
Effects of manipulating SR activity on the expression and phosphorylation of ERK, JNK, and AKT. **(A)** Western blots of p-ERK/ ERK in LaaβH, saline, and CBIO treated rats, and the quantification of p-ERK. **(B)** Western blots of p-JNK/JNK in LaaβH, saline, and CBIO treated rats, and the quantification of p-JNK. **(C)** Western blots of p-AKT/AKT in LaaβH, saline, and CBIO treated rats, and the quantification of p-AKT. *N* = 3 rats in each group. Notice that LaaβH decreased and CBIO increased the phosphorylation of ERK. ^∗∗^*P* < 0.01, ^∗∗∗^*P* < 0.001.

To explore the possible mechanisms underlying the effects of LaaβH and CBIO, we focused on ERK, AKT, and JNK, which are key stress response signals that have been demonstrated to be involved in the development of seizures (Mielke et al., 1999; Dunleavy et al., 2013). Western blotting revealed that the total ERK levels were unchanged in all groups (Figure 6A). However, the expression level of p-ERK significantly increased, starting at 48 h after seizure induction, in rats treated with CBIO compared to rats treated with the saline control (Figure 6A). In contrast, the expression level of p-ERK significantly decreased in rats treated with LaaβH (Figure 6A). Regarding JNK, LaaβH significantly decreased the phosphorylation of JNK (p-JNK), while CBIO had no significant effect on the expression of p-JNK (Figure 6B). Regarding AKT, LaaβH significantly upregulated the expression of p-AKT, while CBIO had no significant effect on the expression of p-AKT (Figure 6C). Together, these data indicated that the phosphorylation of ERK is related to the activity of SR.

### Interfering With the Phosphorylation of ERK Blocks the Effects of SR Manipulation on Seizure Development

**FIGURE 7:**
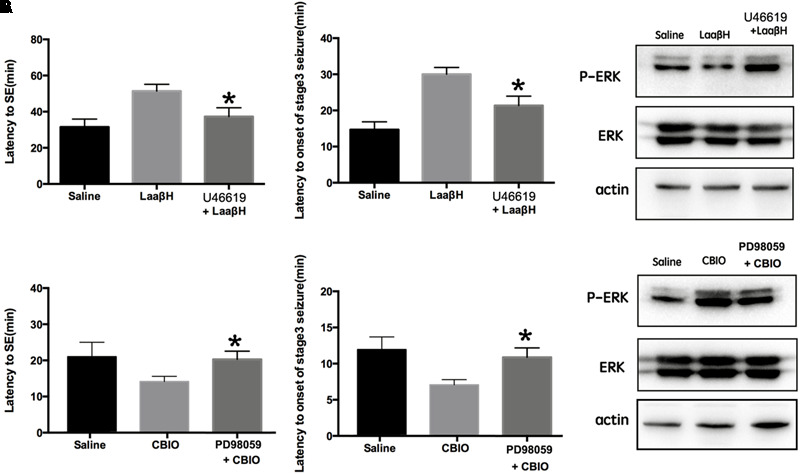
Effects of interfering p-ERK on seizure development in rats treated with D-serine modulators. **(A)** Effects of the ERK activator U46619 on seizure development and p-ERK/ERK expression in rats treated with LaaβH, compared with rats treated with LaaβH alone. *N* = 5 rats per group. **(B)** Effects of the ERK inhibitor PD98059 on seizure development and p-JNK/JNK expression in rats treated with CBIO, compared with rats treated with CBIO alone. *N* = 5 rats per group. Notice that the ERK activator could block the effects of LaaβH and the ERK inhibitor could block the effects of CBIO. ^∗^*P* < 0.05.

It has been reported that the activity of ERK is related to seizure development (Curia et al., 2013; Mohammad Jafari et al., 2018). Because the phosphorylation of ERK is closely related to the expression of SR in the hippocampus, we next tested whether ERK activity was required for the effects of SR manipulation on seizure development. The pretreatment of rats with U46619 (an ERK activator) could significantly block the effects of LaaβH on the latencies to seizure development and to onset of stage 3 seizures (Figure 7A). In contrast, the pretreatment of rats with PD98059, an ERK inhibitor (Jiang et al., 2005), significantly increased the latencies to seizure development and to the onset of stage 3 seizures in CBIO treated rats (Figure 7B). The effects of U46619 and PD98059 on the phosphorylation of ERK were confirmed by Western blotting (Figure 7A,B). These data indicated that the phosphorylation of ERK was an important downstream event of SR during seizure development.

## Discussion

In the present study, we first examined the expression of SR in the hippocampi of rat and humans who experienced seizures. Our results showed that SR expression was primarily increased in the hippocampal astrocytes of epileptic rats and TLE patients. Then, by pharmacologically manipulating the metabolism of D-serine, we investigated the role of D-serine in seizure development. Decreasing D-serine levels prevented the development of seizures, while increasing D-serine levels facilitated seizure occurrence. Furthermore, our Western blotting and behavior analyses showed that the phosphorylation of ERK may be required for the effects of D-serine in seizure development.

In the present study, we adopted the repeated injection of low-dose pilocarpine (50 mg/kg) to induce seizures. This ramping protocol has been shown to reduce mortality after status epilepsy and to better mimic the human TLE (Groticke et al., 2007). Within a few minutes following pilocarpine injection, piloerection, tremor, salivation, diarrhea, immobility, staring, facial automatisms, head nodding, and forelimb clonus were observed in all the animals. Because D-serine is produced from L-serine, we focused on the expression of SR, which is a pyridoxal-50-phosphate (PLP) dependent enzyme that converts L-serine to D-serine (Wolosker et al., 1999). Previous studies using SR knockout mice reported that SR is mainly expressed by neurons and reactive astrocytes (Balu et al., 2014; Li et al., 2018). Our previous study has demonstrated that, upon pilocarpine treatment, the up-regulation of D-serine in neurons peaked at 24 h and then went down (Liu et al., 2009). Our present study focused on the expression of SR in astrocytes 48 h after seizure induction, when reactive astrocytes appear. The differences may be due to the differences between animal species and differences between the observed time points. Our observation that SR was expressed by both astrocytes and neurons was consistent with previous reports (Miya et al., 2008; Martineau et al., 2013). The up-regulation of astrocytic SR in both rats and human patients suggested a conserved response for SR in both rat and human epilepsy. Considering the active roles of hippocampal astrocytes in seizure development (Ryu et al., 2010), these data indicated that the dysfunction in astrocytic SR may be involved in the pathology of seizure development, although the roles of neuronal SR in seizure development is not excluded.

To pharmacologically manipulate the levels of D-serine, we injected two compounds targeting enzymes in the metabolic pathway of D-serine prior to seizure induction directly into lateral ventricles. As an inhibitor of SR, LAAβH can form an aldimine species with PLP and act as a transition state mimetic to decrease the level of D-serine. Our results, showing that LAAβH treatment alleviated the development of seizures, were consistent with previous reports showing that SR knockout could attenuate the expression of seizures (Harai et al., 2012). Because LAAβH has many off-target effects, such as blocking glutamate uptake and inhibiting glycosylasparaginase (Deitmer and Schneider, 1997; Garg et al., 2008; Pande et al., 2017), we then used CBIO, an antagonist of the flavoenzyme D-amino acid oxidase (DAAO), which catalyzes the specific oxidative deamination of neutral D-amino acids, to enhance the levels of D-serine. The increase of DAAO activity in the cerebellum, pons, and medulla oblongata coincides with the decrease of D-serine concentrations in these regions (Strick et al., 2011). The oral or systemic administration of DAAO inhibitors could elevate central D-serine levels (Ferraris et al., 2008; Rais et al., 2012), thereby enhancing NMDAR-mediated functions. Although some reports observed minor effects of CBIO on the levels of D-serine in brain when systemically administered (Hashimoto et al., 2009), our results showed that lateral ventricle injection of CBIO significantly enhanced the levels of D-serine in hippocampus and facilitated the development of seizures. These data indicated that D-serine may be actively involved in seizure occurrences. Manipulating D-serine post-seizure onset may be helpful for preventing seizure reoccurrence, which is worthy of future investigation.

To explore the possible mechanisms underlying the involvement of D-serine in epilepsy, we focused on the stress signaling factors ERK, JNK, and Akt. The results showed that only the phosphorylation of ERK was affected by changes in D-serine levels. A previous study reported that pilocarpine increased ERK activation prior to the induction of seizures (Houser et al., 2008). Our study also revealed that the ERK inhibitor PD98095 and the ERK activator U46619 could effectively block the effects of changes in D-serine levels on seizure development. Therefore, it is possible that ERK signaling is required for the function of D-serine in seizure development. A previous study demonstrated that ERK activation could stimulate NMDA receptor activity (Nateri et al., 2007). It is possible the ERK may be a key downstream signaling molecule that is required for the function of D-serine in increasing neuronal excitability. Together, our data, for the first time, demonstrated that D-serine contributed to the development of seizures via ERK signaling. Specific antagonist of D-serine could be developed for the treatment of epilepsy in the future.

## Author Contributions

TM and YiW performed most experiments and analyzed the data. BC, WZ, LJ, and CS contributed to immunohistochemistry. YaW and YL supervised the projection, analyzed the data, provided financial support, and prepared the manuscript.

## Conflict of Interest Statement

The authors declare that the research was conducted in the absence of any commercial or financial relationships that could be construed as a potential conflict of interest.
