# Corrigendum: D-serine contributes to seizure development via ERK signaling

**DOI:** 10.3389/fnins.2025.1541189

**Published:** 2025-02-17

**Authors:** Tie Ma, Yin Wu, Beibei Chen, Wenjuan Zhang, Lang Jin, Chenxi Shen, Yazhou Wang, Yonghong Liu

**Affiliations:** ^1^Department of Neurology, Xijing Hospital, Air Force Military Medical University, Xi'an, China; ^2^Department of Neurology, The Seventh Medical Center of PLA General Hospital, Beijing, China; ^3^Department of Pharmacy, Xi'an High-tech Hospital, Xi'an, China; ^4^Department of Neurobiology and Institute of Neurosciences, School of Basic Medicine, Air Force Medical University, Xi'an, China

**Keywords:** d-serine, serine racemase, astrocyte, epilepsy, hippocampus

In the published article, there was an error in Figure 5A as published. Duplicate images were mistakenly displayed in the inserts 1 and 2 of Figure 5A. The corrected Figure 5 and its caption appear below.

**Figure 5:**
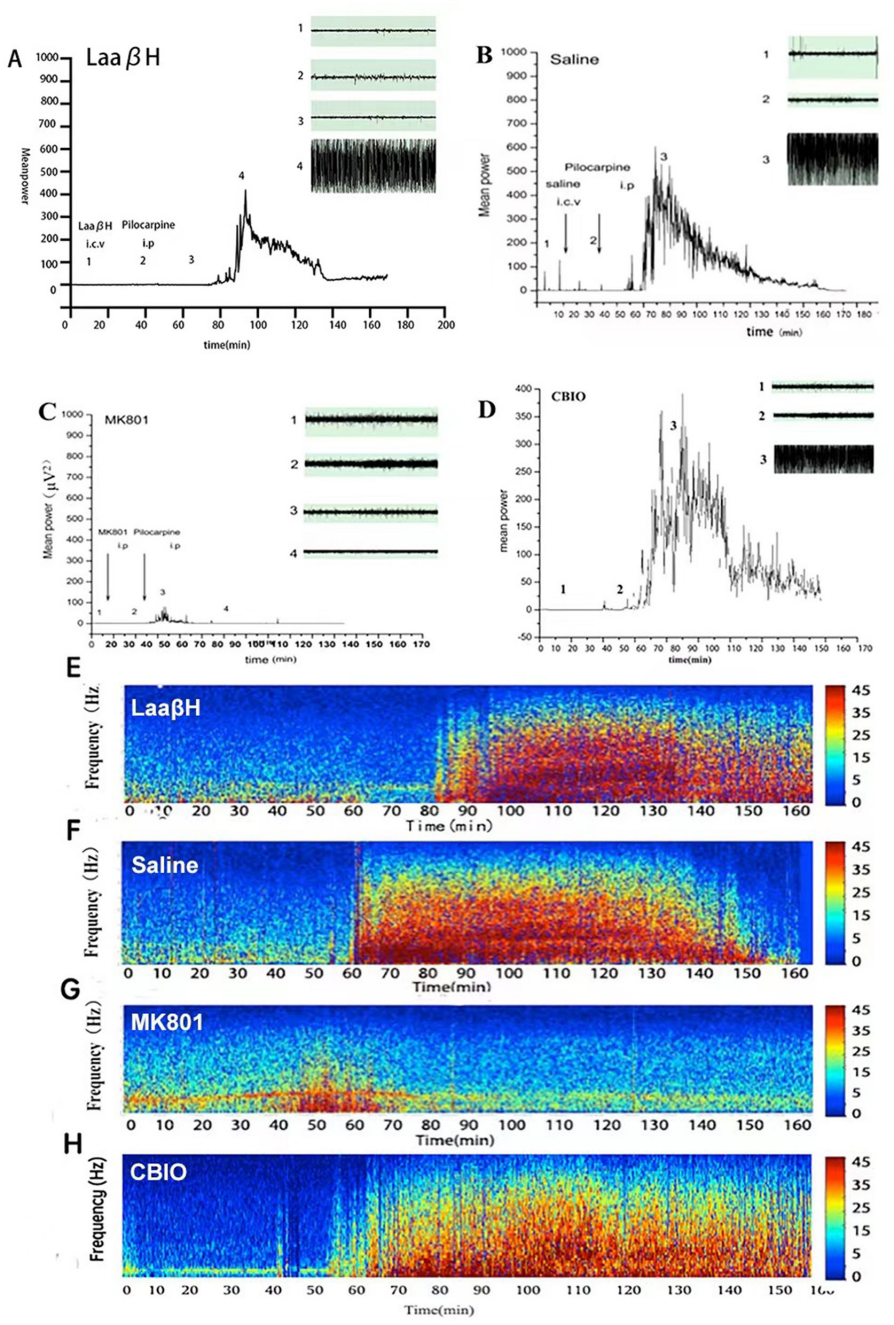
Effects of MK801, LaaβH and CBIO on EEG recordings. **(A–D)** Mean power of EEG recordings in rats treated with LaaβH **(A)**, saline **(B)**, MK801**(C)** and CBIO **(D)**. **(E–H)** Representative frequency images of EEG recordings in rats treated with LaaβH **(E)**, saline **(F)**, MK801 **(G)** and CBIO **(H)**. *N* = 7–9 rats per group. Compared with the saline control, LaaβH could prolong the onset of seizure occurrence and reduce the mean power of the EEG, while CBIO could shorten the onset of seizure induction and increase the mean power of the EEG.

The authors apologize for this error and state that this does not change the scientific conclusions of the article in any way. The original article has been updated.

