# Supplementary figures and images for: D-Serine Contributes to Seizure Development via ERK Signaling
Source: Front Neurosci. 2019 Mar 26;13:254. doi: 10.3389/fnins.2019.00254 (PMC6443828; doi:10.3389/fnins.2019.00254)

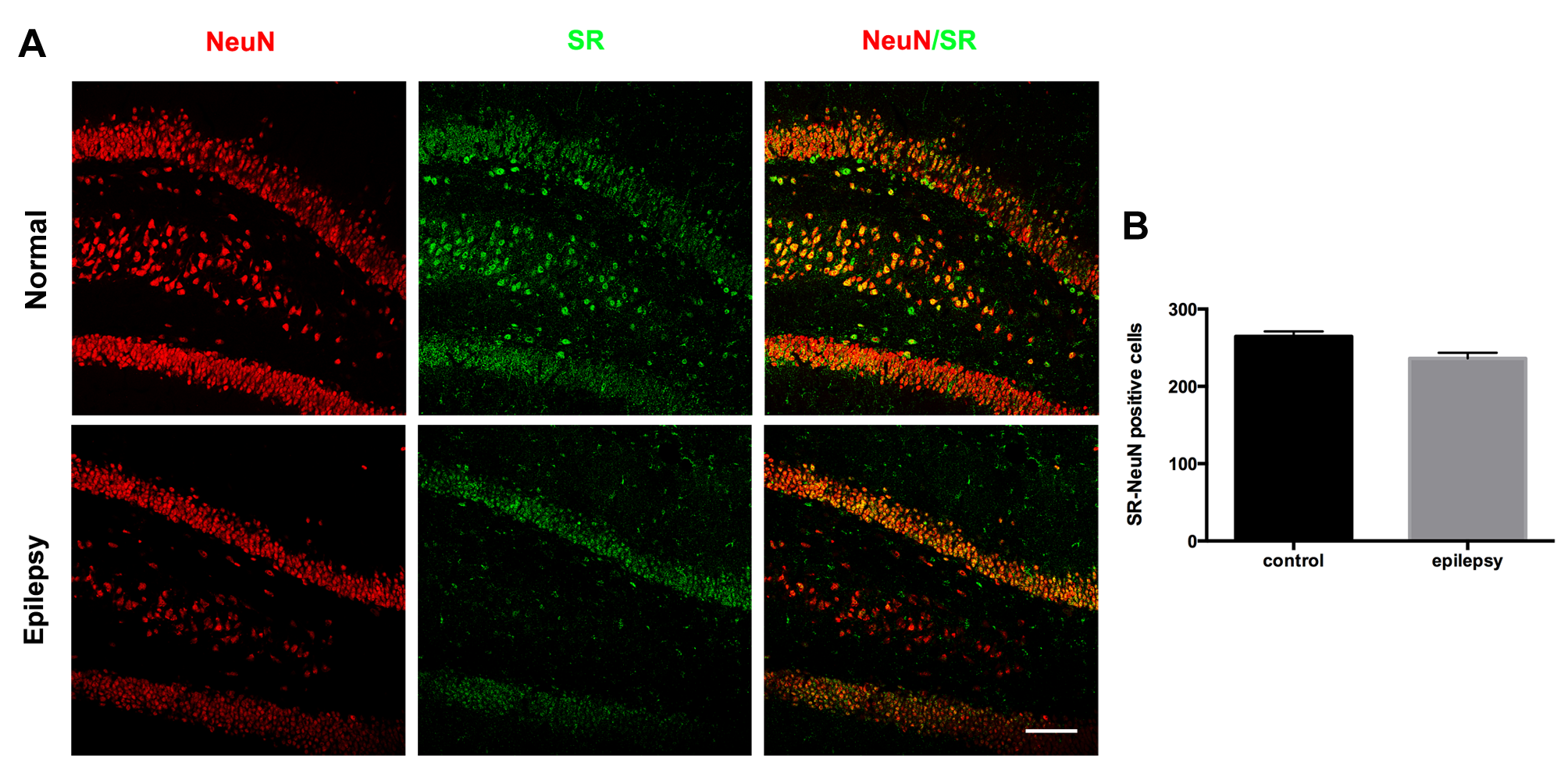

Supplement: Supplementary file 1 [file Image_1.tif]

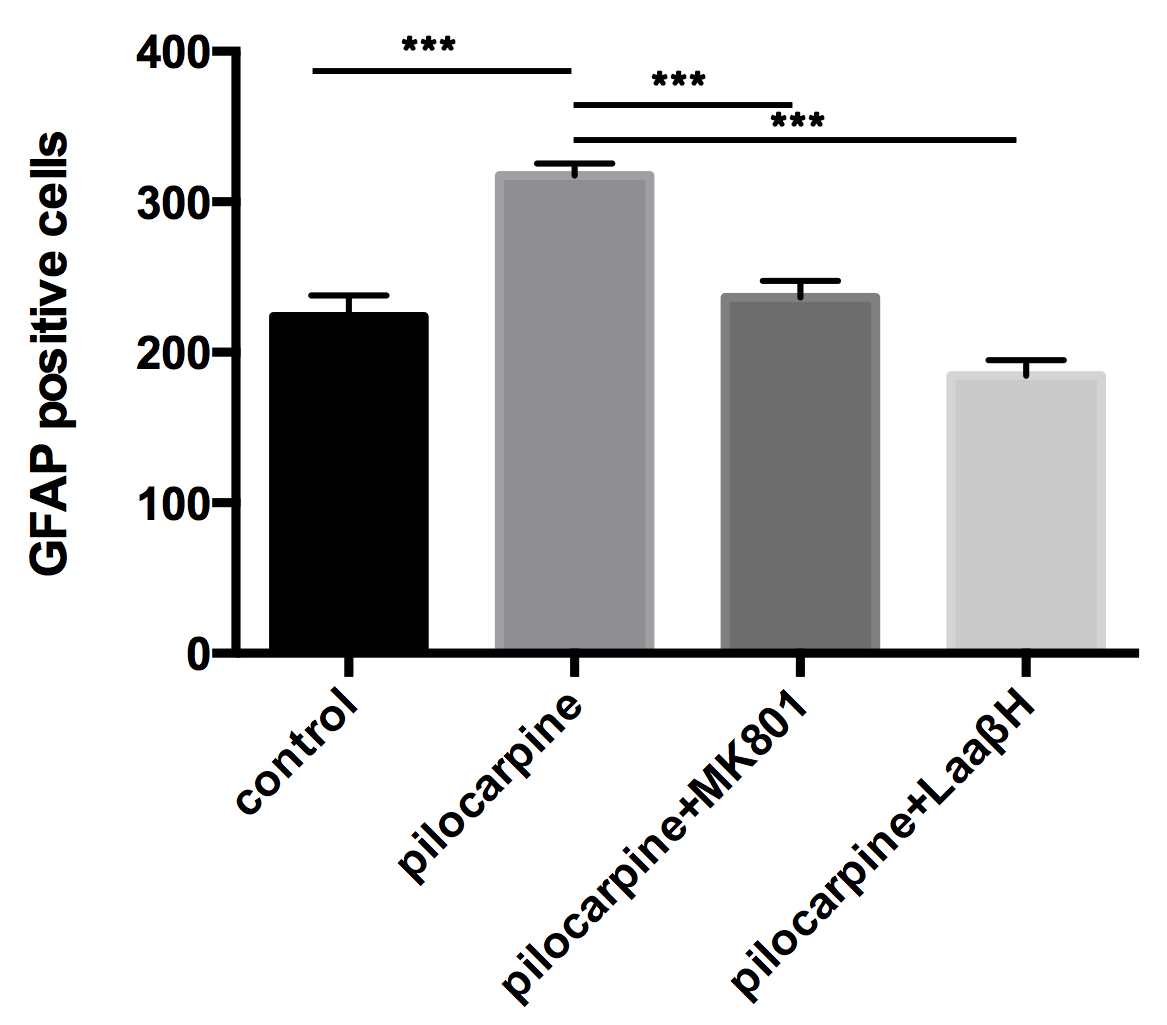

Supplement: Supplementary file 2 [file Image_2.tif]

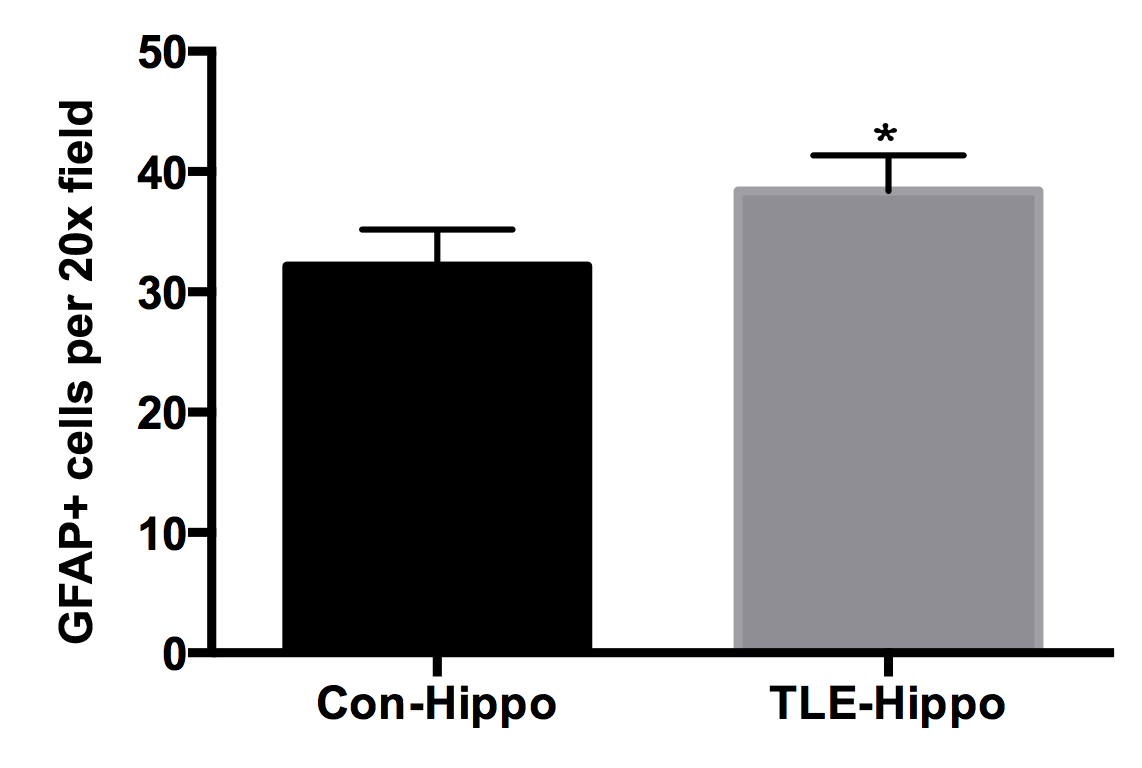

Supplement: Supplementary file 3 [file Image_3.tiff]

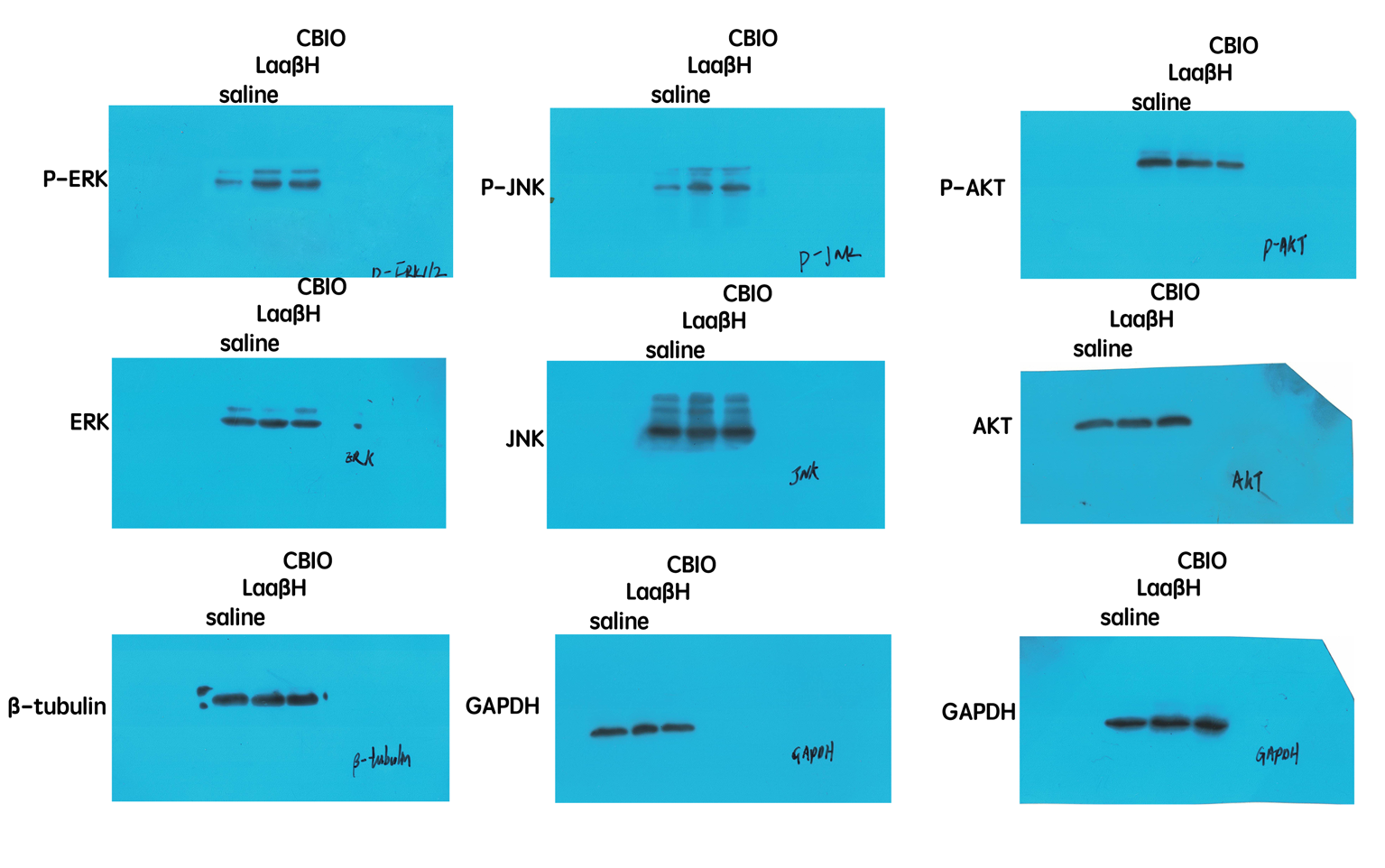

Supplement: Supplementary file 4 [file Image_4.tif]

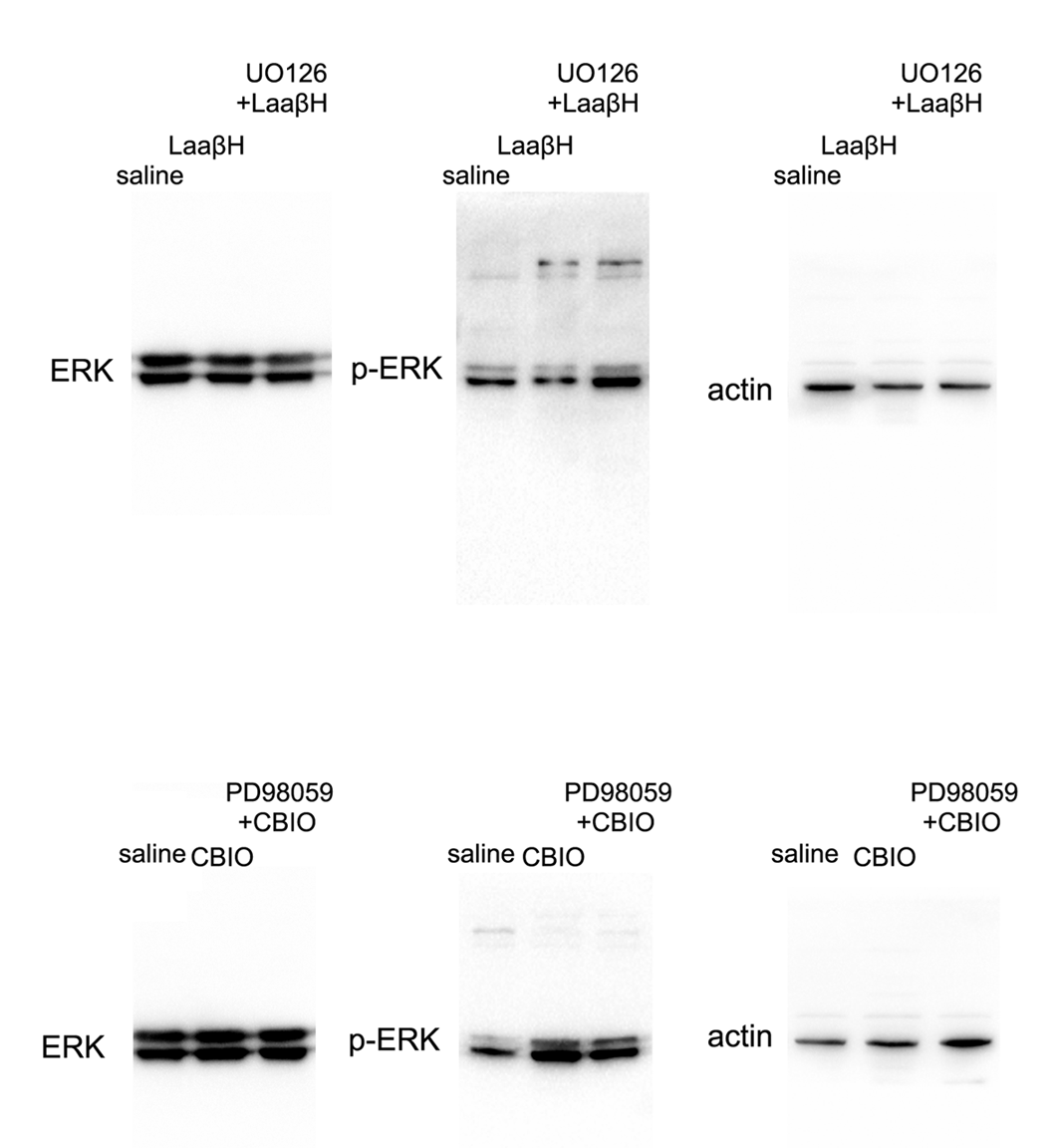

Supplement: Supplementary file 5 [file Image_5.tif]
