# Supplementary material for: D-Serine Contributes to Seizure Development via ERK Signaling
Source: Front Neurosci. 2019 Mar 26;13:254. doi: 10.3389/fnins.2019.00254 (PMC6443828; doi:10.3389/fnins.2019.00254)
Supplement: Supplementary file 6 [file Table_1.docx]

**Title:** Contribution of D-Serine to seizure development via ERK signaling

**Authors:** Tie Ma^1,2*^, Yin Wu^3*^, Beibei Chen^1^, Wenjuan Zhang^1^, Lang Jin^1^, Chenxi Shen^1^, Ya-Zhou Wang^4#^, Yonghong Liu^1#^

**Supplementary information**

**Supplementary Figure-1. Expression of SR in neurons after seizure-induction in rats.**

(A) Double-immunostaining of NeuN and SR in normal and seizure-attacked rats. (B) Quantification of SR-positive neurons. Notice that there is no significant difference of SR-positive neurons between normal and seizure-attacked rats. Bar=50μm.

**Supplementary Figure-2. Quantification of GFAP-positive cells in Figure-1.**

**Supplementary Figure-3. Quantification of GFAP-positive cells in Figure-2.**

**Supplementary Figure-4. Full-gel images of Figure-6.**

**Supplementary Figure-5. Full-gel images of Figure-7.**
